# Optimization of protocol design: a path to efficient, lower cost clinical trial execution

**DOI:** 10.4155/fso.15.89

**Published:** 2016-01-12

**Authors:** Marina A Malikova

**Affiliations:** 1 Department of Surgery, Boston University, 88 East Newton street, Collamore building, Department of Surgery, Boston, MA 02118, USA

**Keywords:** managing complexity, mitigation of fiscal risks, optimization of study protocol design

## Abstract

Managing clinical trials requires strategic planning and efficient execution. In order to achieve a timely delivery of important clinical trials’ outcomes, it is useful to establish standardized trial management guidelines and develop robust scoring methodology for evaluation of study protocol complexity. This review will explore the challenges clinical teams face in developing protocols to ensure that the right patients are enrolled and the right data are collected to demonstrate that a drug is safe and efficacious, while managing study costs and study complexity based on proposed comprehensive scoring model. Key factors to consider when developing protocols and techniques to minimize complexity will be discussed. A methodology to identify processes at planning phase, approaches to increase fiscal return and mitigate fiscal compliance risk for clinical trials will be addressed.

**Figure 1.**
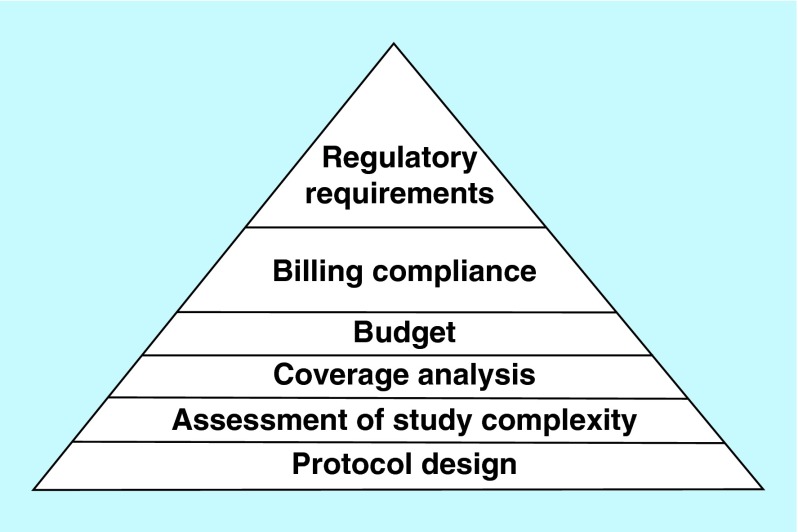
**Project planning: managing complexity of study protocol, ensuring billing compliance and adherence to current regulatory requirements.** Optimizing protocol design, performing complexity assessment of study protocol and procedures at planning phase of a trial will allow for more accurate coverage analysis, cost estimation/budgeting, mitigation of fiscal risks and ensure compliance with current regulatory requirements that will warrant successful trial execution with adequate allocation of resources needed.

**Figure 2.**
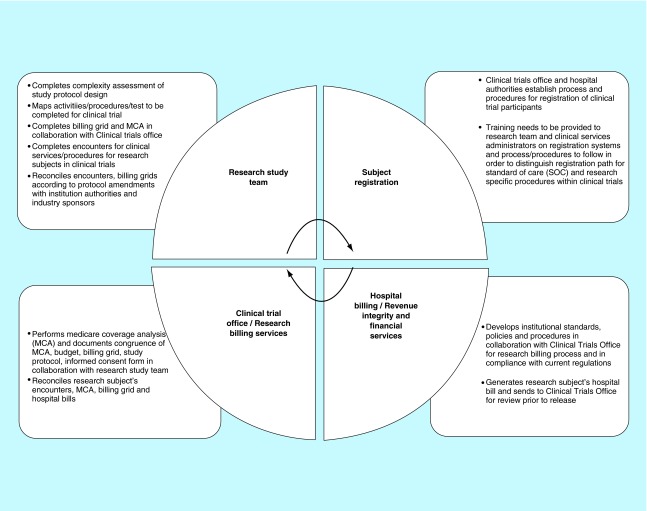
**Organizational process defining roles and responsibilities between research teams, organizational authorities and sponsors in development and implementation of billing process for clinical trials.** Development and implementation of billing process for clinical trials should include establishment of billing compliance framework, standard operating procedures and process defining roles and responsibilities between research teams, organizational authorities and sponsors.

## Background

Although technological innovations have shortened drug discovery and preclinical development phases, the clinical testing phase has not made similar progress [1,2]. Costs associated with the implementation of clinical trials have become an increasingly important issue, yet little has been done to develop cost reduction approaches and organize efforts to improve clinical study efficiency and performance [1–3].

As regulatory landscape changes and requirements for safety and efficacy become more stringent, life sciences companies are looking for new adaptive approaches to shorten duration of clinical phases of drug development and implement new ways to decrease study protocol complexity [4]. Clinical forecasting in drug development is critical to establish key safety and efficacy parameters, improve trial designs, select appropriate target populations, control duration and costs [4]. Several research groups reported that complex clinical study protocols lead to difficulties at implementation phase, cause delays in enrollment completion and as a result prolonged duration of clinical testing phase contributes to the rise of drug development costs [5,6]. In response to regulatory changes, drug developers are attempting to find new pragmatic strategies to contain costs by proactively addressing study protocol complexity and optimizing study design [5–7].

Currently, many organizations involved in biomedical products development are trying to benchmark their own internal study design practices against published data, develop robust metrics to assess study complexity and optimize protocol design [5,6,8]. Benchmarking offers an opportunity for organizations involved in the process of developing biomedical products to really understand how their study protocol designs compare to general industry practice [5–7]. For example, do they have too many end points and/or too many assessments? Are these end points necessary to obtain regulatory approvals or just exploratory in nature? Are study procedures too complex to execute?

We have attempted to create a methodology that allows to assess study protocol complexity, raise concerns upfront regarding study procedures, discuss primary and secondary data end points (outcomes), which need to be obtained for a specific study, estimate clinical research team workload and allocate resources accordingly to ensure successful execution. This approach is aimed to assess feasibility of a study to be conducted at particular institution (i.e., assess availability of clinical services, equipment needed to collect outcomes, etc.), streamline tasks during execution phase, decrease redundancies, eliminate unnecessary procedures (especially invasive ones) and concentrate on what is really important to deliver high-quality results, while increasing efficiency of a study team executing study-related tasks.

This proactive strategy is aimed to engage investigators from clinical sites (academic hospitals or private practices) in earlier discussion of a study protocol with the industry sponsors who often design clinical protocols on their own. Investigators from clinical sites can bring unique perspective, raise any potential issues regarding implementation phase, and make recommendations regarding which procedures are best practices in order to attain certain outcomes. Open dialogue between industry sponsors and investigators upfront can decrease number of procedures needed to be done within a study, decrease number of protocol amendments in the future, ensure higher accrual rates of study subjects and make implementation phase much more efficient.

It is important to receive potential clinical sites’ feedback regarding protocol complexity and feasibility of study procedures. If investigators from clinical sites are presented with opportunity to collaborate with sponsors at the earlier stages of drug/device development plan and get involved at the earlier stages of protocol development, they can provide valuable feedback to the sponsors based on their clinical and research experience in a relevant therapeutic field. There is growing realization within industry and clinical sites that the optimization of protocol design is an absolute necessity for their long-term success [6–8].

Among factors influencing study quality and integrity are well-designed study protocol and Data Safety Monitoring Plan. The Data Safety Monitoring Plan should be study specific and include the following: mix of centralized and on-site monitoring practices, utilization of electronic Case Report Forms versus traditional hard copy Case Report Forms, clear study objectives, critical efficacy and safety data, designated roles and responsibilities of qualified and trained investigators, study staff and monitors.

Developers of new biomedical products must review key protocol design parameters and define critical data and processes at study planning stage as critical for successful trial implementation, reduction of cost and adherence to timelines. Typically, the following data/processes are among ones which are considered universal for a clinical trial of any size and/or phase:

Data supporting primary and secondary objectives;
Data critical to subject safety: serious adverse events and events leading to investigational drug/device discontinuation;
Data critical to trial design and statistical end points (i.e., safety and/or efficacy primary study outcomes; secondary outcomes such as quality of life parameters, socioeconomic parameters, cost utilization data, etc.);
Adherence to eligibility;
Adherence to study protocol and procedures;
Informed consent process (should be conducted prior to any study related procedures);
Documentation of administration of investigational agent or treatment procedures.

Some organizations are also looking specifically at reducing the number of protocol amendments that they typically must implement.

## Assessment of study design complexity: study protocol, processes, procedures and scoring model

The goal in creating the criteria for a trial complexity assessment was to develop an easy to follow and implement standardized model assigning a representative value/score to trial parameters deemed to involve increased effort at the participating sites. These parameters are not designed to describe every detail of a trial, but rather they were selected as those most likely to identify the most time-consuming and complex work for accruing participants. The impact of trial complexity on the sponsor, and/or collaborator (i.e., CROs, other third party vendors such as centralized core laboratories, etc.) is not included in this model, as the focus is on the work load at the participating clinical site. Each parameter was ranked according to three categories based on its level of complexity: routine, moderate and high. Feedback on this study protocol complexity scoring model was further gained from administrative staff, research nurses, coordinators, clinical research associates and investigators. Studies deemed ‘complex’ based on the ten parameters described in the complexity model (see Table 1 for more specific details) may be eligible to receive additional institutional funds/resources allocation, when available, and/or adjustments have to be taken into consideration when negotiating trial budgets with the sponsors.

### Parameter # 1: number of study arms/study groups

This parameter is assessed based on number of study groups required to implement study design. Categories for the number of study arms based on study protocol design:

Level 0 (routine): one to two study arms;
Level 1 (moderate): three to four study arms;
Level 2 (high): more than four study arms and/or formal crossover or factorial study design.

### Parameter # 2: informed consent process

This parameter is assessed by estimating the extent of effort it would take to describe a study to a potential research subject and obtain informed consent. Study protocols involving molecular markers or targeted therapies (i.e., gene therapy, vaccines, growth factors, etc.) can make the consenting process more elaborate. Consent process length was not included in scoring model due to the simple fact that length does not always correlates with content of activity to be performed and/or complexity. However, in order to capture the increased effort required of the site to provide an adequate informed consent process, the complexity of the randomization/registration process is incorporated into the levels below as well as considered as a separate parameter. Also, the issue of translation of the informed consent into other languages and utilization of certified translators for informed consent process is addressed in Parameter # 3 ‘enrollment feasibility/study population’.

### Parameter # 3: enrollment feasibility/study population

For this parameter, the level of complexity will correspond to the difficulty in trial participant identification; highly selective eligibility criteria leading to high ‘screening-failure’ rates; or the scarcity of trial population (i.e., rare tumor types, uncommon disease or condition) with the higher level of complexity. Study that includes vulnerable populations, such as elderly, pregnant women, minority groups and terminally ill patients will require special provisions for protection of vulnerable populations, and additional recruitment efforts will be needed. Due to the subjectivity of this parameter, justification will be required for an assignment of ‘moderate’ or ‘high’ level.

### Parameter # 4: registration of study participants & randomization process

This parameter should be assessed by site personnel based on the effects of the study design on the site logistics and effort required to register study participant and/or perform randomization procedures and in conjunction with Parameter # 2: informed consent process, depending on time allowed by study protocol between consent and randomization procedures.

### Parameter # 5: nature of investigational product & complexity of administration

The nature of investigational product (IP), delivery route (intravenous, oral or topical) and complexity of IP administration (single vs multiple times of administration during treatment phase, acute vs chronic indications) will affect effort allocation from study team and may require additional institutional approvals to be obtained. In earlier phase of drug development, with more uncertain safety profile of IP more toxicities are anticipated, and more experienced with earlier phases of drug development investigators and team members are preferred to be engaged. Certain therapeutic indications are more complex by nature (i.e., gene therapy, growth factors, vaccines, etc.) and they pose high risks and safety concerns. Typically for these IPs, data safety monitoring plans are required to be developed, reviewed and approved by Institutional Biosafety Committee (IBC).

For high-risk studies (i.e., Phase I – ‘first in human’ trial, gene therapy studies with complex treatment regimens, high potential for toxicities and complicated investigational product handling procedures), personnel trained and experienced with Phase I study and/or specific therapeutic class of study product are preferred to be involved in execution phase to mitigate risks. Additional effort for training/credentialing and education of site personnel as well as more intensive involvement of pharmaceutical company for support and oversight will be required.

Other special situations include Institutional Radiological Safety Committee (IRSC) approvals for investigational products based on radioactive isotopes (new radiocontrast agent, new use of prior approved radioactive agent with significant changes to currently approved dose/regimen/route of administration for a specific disease/indication and not currently utilized as part of routine clinical care), and/or if required by study protocol as part of diagnostic or interventional study procedures beyond routine clinical care (i.e., additional x-rays to assess study outcomes, new type of experimental positron emission tomography scan, etc.). Additional exposure to radiation needs to be assessed by IRSC and approved prior to implementation, if utilized in clinical study designs, IRSC will need to make determination based on scientific rationale provided and safety assessments.

Personnel handling administration of radioactive isotopes as part of study treatment and/or study-specific procedures need to be credentialed, trained and experienced with such products/procedures. Additional oversight and training to be provided by industry sponsor developing/testing radioactive product and/or utilizing isotopes as part of study protocol procedures will be recommended. This needs to be addressed in conjunction with Parameter 10A (correlative pathology/imaging) to adequately allocate resources for a specific study design implementation that involves imaging procedures with radioactive agents.

### Parameter # 6: length of investigational treatment phase

Length of treatment phase should be considered. However, as it varies so drastically, it can be difficult to incorporate it in scoring model. As this parameter is so difficult to quantify, taking into consideration, particular study protocols will be critical and modifiers can be used for more accurate estimation of complexity.

#### Modifiers for length of investigational treatment

Level 0 (routine care/standard):

Regimens with a defined number of cycles;
Routine or standard therapy (i.e., 5 years of tamoxifen or aromatase inhibitors for treatment of breast cancer).

Level 1 (moderate):

Lengthy study with treatment until progression (i.e., cycles of treatment are not a defined number), individual assessments/adjustment of dosage and/or regimen are required;
Long period of hormonal, chemotherapy or standard maintenance therapy for chronic diseases/indications/conditions, in addition to investigational agents.

Level 2 (high):

Extended administration of investigational agent by sites, such as longer than 6 months (i.e., chronic indications such as rheumatoid arthritis, blood disorders, certain types of cancer, diabetes, hypertension, etc.).

### Parameter # 7: study teams/study staff

This parameter addresses personnel who need to be involved in order to execute clinical study according to proposed protocol design (i.e., Investigational Drug Services [IPS], pharmacists, radiologists, cardiologists, pathologists, translational scientists, biostatistician, etc.). In order to quantify this parameter, the more disciplines needed to coordinate and implement multidisciplinary trials, the more complex trial management becomes for sites. In addition, trials that require sites to engage personnel in disciplines that previously or typically did not participate in clinical trials may be scored as high complexity due to lack of experience and additional time and effort allocation that will be required to onboard clinical specialty/services/team with no prior exposure to clinical research.

### Parameter # 8: data collection complexity

As a protocol becomes more complex, so does the data collection process. Complex regulatory submissions for trials involving biologics and combination products must be considered in scoring model.

### Parameter # 9: follow-up requirements

Length of time required by study protocol for follow-up phase may also be taken into consideration. However, common discrepancy among actual versus planned study subjects’ follow-ups must be taken into consideration (i.e., due to toxicity, serious adverse events, etc.).

### Parameter # 10A & B: ancillary studies

Ancillary studies and/or utilization of ancillary services (i.e., core pathology laboratory, various radiology/imaging tests, electrocardiograms [EKGs], etc.) must be included in the complexity assessment model due to the increased work required from the site personnel to coordinate and/or perform these additional assessments. Also, additional costs for technical and professional charges associated with ancillary studies as related to the study design should be addressed with coverage analysis and included in the budget at planning/start-up phase in order to accurately estimate study costs and mitigate fiscal risks.

In order to standardize the scoring process, the two different categories of the ancillary studies Parameters 10A (correlative pathology/imaging) and 10B (quality of life [QoL], questionnaires, socio-economical assessments, health services utilization, etc.) should be considered to be included in scoring model.

A protocol that has both types of ancillary studies has the potential to score up to four points for trial complexity (0–2 for Part A; 0–2 for Part B). Only mandatory by study protocol and incorporated as required parameter in the study design correlative imaging/pathology studies should be factored into the scoring of this parameter. The justification of scores, especially if multiple additional factors are considered when scoring, is needed (i.e., renal clearance test is required, if contrast is used in imaging studies; pregnancy test is required, if radiological probe is used for tests, etc.).

## Study protocol complexity score applications for traditional & adaptive study protocol designs

For each parameter described above and in the Table, the following scoring method applies: 0 points for ‘Routine or Standard’, 1 point for ‘Moderate’ and 2 points for ‘High’ rating. The rating for each parameter should be assigned according to study protocol design and resources needed to conduct a study. Specifically, Parameter # 10 may contribute two sets of complexity scores if a trial has the two different categories of ancillary studies based on the ancillary studies level of assessments referred to in the Table as Parameter # 10A* (correlative science/lab/imaging) and Parameter 10B** (QoL, or health services utilization). Therefore, a protocol that has both types of ancillary studies, correlative science and QoL, has the potential to score up to 4 points (0–2 for correlative and 0–2 for QoL), depending upon which modifier is used for ancillary studies required by protocol design. Once the ten parameters are rated for a specific protocol design, their associated scores can be added up and the total overall complexity score can be assigned to a clinical trial as an ultimate rating of a study protocol design as ‘Routine/Standard’, ‘Moderate’ or ‘High’ based on that final score.

Typically, study protocols that include simple study procedures and topical investigational product administration (no systemic exposure) rank as ‘Routine/Standard’ to ‘Moderate’ in complexity. Studies with complex designs and high level of anticipated toxicities (i.e., early phases of cancer trials with novel chemotherapeutic investigational products, gene therapy, etc.) rank as ‘High’ in complexity score and will require more resources at execution phase.

In addition, formal interim analyses can be conducted as part of an adaptive trial design to assess data collected in clinical trials. Typically, interim analysis is performed in trials that have a Data Safety Monitoring Board/Committee (DSMB/DSMC), longer duration of recruitment (i.e., chronic indications and/or long-term follow-up is required) and potentially serious outcomes with complex study protocol designs for life-threatening diseases such as cancer trials. The results of these analyses contribute to decision-making process of putting study on clinical hold, if findings of interim analysis revealed safety concerns such as increased rate of serious, unexpected and related to investigational product or study procedures events; high rate of drop outs from the study due to adverse events; pending further investigation this hold can be lifted. Also results of an interim analyses can be a guideline that helps inform investigators whether the trial should be continued as originally designed, modified or terminated earlier than intended for clinical benefit (i.e., efficacy of investigational drug/device is established), harm/risks (i.e., high level of serious adverse events proven to have strong association with investigational product) or futility reasons (i.e., lack of efficacy). Often criteria for stopping a trial for safety reasons are different from those for benefit (i.e., efficacy of investigational product is confirmed by interim analysis results as compared with control treatment) and may not utilize a formal statistical criteria. Stopping for futility based on interim analysis results occurs in instances where, if the study were to continue, it is very unlikely that an important effect would be seen (i.e., low chance of rejecting null hypothesis). Apart from providing basis for stopping guidelines, planned interim analyses can be used in adaptive trial design for sample size adjustments, change to the proportion of subjects’ allocation to study groups and amendments of eligibility criteria.

Complexity parameters can be reviewed again at execution phase (i.e., approximately at 15–25% of planned enrollment at participating site) and scoring of a study protocol can be reassessed for revisions, if needed, after this model is implemented and based on specific experience as it develops during execution phase of a particular trial.

## Project planning: managing complexity of study protocol, ensuring billing compliance

A clinical trial shares many features with any other type of business project as defined in the field of project management [9]. These features include the following: a clear objective aimed to bring about change, requiring a team, a set time scale, defined resources to achieve its objective, tasks that need to be completed according to a prespecified standard and current regulations. Planning for completion of the various tasks via a detailed timeline and scope of work is crucial for future success of a clinical trial (Figure 1) [9,10].

As transition occurs from planning to implementation phase, the amount of work and number of players involved increases significantly, and management of multiple aspects of the study become critical in ensuring adherence to an established timeline.

Once the plan is created, a project manager should manage the study according to study protocol, project scope and operate within established timeline. For each study plan, careful consideration needs to be given to the timeline with realistic milestones and assessment of study protocol complexity. Developing a standard rating scale/scoring model to evaluate clinical trial complexity and applying this mechanism to facilitate workload of study teams will allow to navigate through complex study procedures, ensure adherence to study protocol and mitigate risks with billing compliance (Figure 1).

Academic medical centers and private practices participating in clinical trials face new challenges with billing compliance under more stringent regulatory requirements [11–13]. It is imperative to perform Medicare Coverage analysis upfront to establish billing framework and distinguish costs that will be paid by the sponsor of a clinical trial versus costs that can be billed to medical insurance [12,13].

According to Clinical Trial Policy National Coverage Determination (NCD) issued on 9th July 2007 by Centers for Medicare and Medicaid Services (CMS), Medicare, one of the major insurance carriers/provider established by US government, covers the routine costs of qualifying clinical trials; and other reasonable and necessary services used to diagnose and/or treat complications resulting directly from participation in all qualified clinical trials [14,15]. Currently, this is a standard policy established by governmental agency based on which insurance companies, new biomedical product developers and medical centers make clinical trials coverage decisions. Lack of adherence to this policy and failure to perform coverage analysis upfront can lead to billing errors and increase fiscal risks of organizations conducting clinical trials that are obligated to follow federal regulations and operate accordingly with current billing compliance requirements [14,15].

## Determination of routine costs versus research-related costs in clinical trials in accordance with regulatory requirements & study protocol design

According to Clinical Trial National Coverage Determination (NCD) policy, routine costs include all items and ancillary services that would normally occur as part of the patient's care outside of a clinical trial [15,16]. Costs associated with the prevention, diagnosis and/or treatment of complications directly resulting from participation in clinical trials are also covered under NCD [16].

Determination of routine costs versus research-related costs in clinical trials in accordance with regulatory requirements and study protocol design must be made prior to initiating clinical study. In order to receive reimbursements from insurance for costs accumulated for services, procedures conducted as part of clinical trial, a study must meet the following main three requirements under the NCD to receive Medicare coverage for routine costs [16]:

The main objective or purpose of the trial must be the evaluation of an item or service that falls within a Medicare benefit category [16];
The trial must not be designed exclusively to test toxicity or disease pathophysiology. It must have therapeutic intent (i.e., intent-to-treat design of a clinical study protocol by design) [16];
Trials of therapeutic interventions must enroll patients with diagnosed disease/condition rather than healthy volunteers. Trials of diagnostic interventions may enroll healthy patients to have a proper control group [16].

However, meeting these three main requirements does not guarantee Medicare coverage approval of routine costs for services/tests/procedures conducted within clinical trials. Additional seven desirable characteristics of qualified clinical trials must be addressed by applicants to CMS. Among them are the following criteria as outlined in NCD policies and guidelines [16]:

The main goal/objective/purpose of the trial must be to test whether the intervention potentially improves the participant's health outcomes (i.e., study protocol must incorporate the ‘intent-to-treat’ by design);
The trial must be well supported by currently available scientific, medical information, or it must be intended to clarify or establish the health outcomes of interventions already in common clinical use;
The trial must not unjustifiably duplicate existing studies. The rationale for new proposed study must be provided in comparison with prior conducted studies;
The trial design must be appropriate to answer the research question being asked in the trial;
The trial must be sponsored by a credible organization or individual qualified, experienced and trained of executing the proposed trial successfully;
The trial must be conducted in compliance with federal regulations relating to the protection of human subjects;
All aspects of the trial must be conducted according to the appropriate regulatory standards and have scientific integrity.

Clinical trials that meet all qualifying criteria will receive Medicare coverage of routine costs after the trial's lead principal investigator or institutional authorities on his/her behalf certify that the trial meets the criteria. This process will require the principal investigator to enroll the trial in a Medicare clinical trials registry.

Some clinical trials are automatically qualified to receive Medicare coverage of their routine costs because they have been ‘deemed’ to be highly likely to have the above-listed seven desirable characteristics of clinical trial. Such trials are typically federally funded trials, studies conducted under an investigational new drug application and drug trials that are exempt from having an investigational new drug application under 21 CFR 312.2 (b) [1,16]. The definition of routine costs requires careful examination of a study protocol design by leading principal investigator in accordance with institutional hospital policies/guidelines for standard of care, clinical trial agreement established between clinical research sites and industry sponsors. The decision for routine clinical costs to be ‘billable’ to insurance (i.e., Medicare) is ultimately made by governmental agencies such as CMS in the USA.

The compensation for injury language, if determined to be related to participating in a clinical trial, free of charge services or any payments to research subjects for completed study-specific procedures/study visits stated as such in the informed consent form must be examined carefully and made consistent with the language in corresponding section of clinical trial agreement, clinical trial budget and the billing grid.

## Establishing billing compliance framework, standards & procedures

Development of comprehensive billing compliance policies and guidelines can help investigators and organizations participating in clinical research to establish standardized processes to improve billing compliance, mitigate fiscal risks and achieve better adherence to current regulatory requirements.

In an attempt to adopt federal regulations and establish standardized practices, we follow general process at start-up phase while evaluating a new clinical trial and carry it over further to implementation phase (Figure 2). This process highlights our current organizational practices, and defines roles and responsibilities as shared between research team, organizational authorities and sponsors in development and implementation of billing process for clinical trials (Figure 2):

In collaboration with principal investigator, clinical study industry-sponsor and organizational authorities analyze study protocol design and determine procedures/services/activities that need to be performed according to specific study;
Create study billing grid for research-related services for each study visit in accordance with study protocol prior to enrolling participants into a research study;
Proactively seek feedback on study protocol design and billing grid from all parties that will be involved in implementation of study design and administrative tasks (i.e., consult hospital ancillary services, laboratories that may provide services to research subjects, which will be enrolled in a potential study; cross-check with institutional authorities – Revenue Billing Services at the hospital, Clinical Trial Office for current institutional billing compliance policies, practices and established standards);
Perform Medicare Coverage Analysis and document congruence of Medicare Coverage Analysis, budget, billing grid, study protocol, informed consent form and Clinical Trial Agreement;
Identify services/procedures/tests as standard-of-care (SOC) or routine clinical care versus research-related study when ordering tests and/or providing services. Note that definition of standard of care (routine clinical care) can vary depending on state, regional or country requirements for global trials;
Conduct discussions with organization representatives and industry sponsor through open communications upfront (prior to initiating clinical trial and starting enrollment of study participants) to determine who are the potential payers for activities and/or services performed within clinical trial;
Establish quality controls to determine if services are appropriately coded to SOC and/or research accounts for the registration and tracking of all research subjects for clinical trial and in compliance with current institutional policies and procedures for clinical trials billing, participant registration and expenditure tracking;
Develop a consistent approach throughout the organization that includes personnel who are trained in registering for research in order to capture both SOC and study-related services/charges; reconcile bills/invoices according to the billing grid before submission to Medicare (in the USA) or insurance companies/governmental healthcare services in other countries, depending upon in which state/region/country the trial is conducted.

Inconsistencies in developing billing grids, variations in clinical trial participants scheduling/registration processes and lack of a consistent and centralized reconciliation process can lead to governmental agencies putting clinical trial on hold for billing noncompliance, audits, fines, negative publicity, false claims (wrongful billing practices), damage to the institution's and/or principal investigator's reputation [12,14]. In order to mitigate these risks, organizational policies/guidelines establishing standards for billing compliance should be implemented and followed by trained research personnel and institutional administrators.

## Conclusion & future perspective

Complexity rating models provide clinical research organizations and medical centers with an objective method of quantifying clinical trials activity on the basis of study protocol design and complexity. The main limitation of proposed study protocol complexity scoring model is that recommendations really fitting to every possible trial setting are difficult to make, but on the other hand general recommendations often lack specificity. We have attempted to develop a comprehensive scoring system with multiple complexity assessment parameters that are common for major therapeutic indications and can be utilized to assess study protocol designs of different phases for various clinical trials throughout multiple phases of project's life cycle. With consistent application of complexity scoring model, clinical sites can manage staffing objectively, ensure adherence to study protocol and procedures, effectively allocate resource and mitigate billing compliance risks via performing coverage analysis upfront and in conjunction of study protocol design and complexity.

**Table 1.** **Clinical study protocol complexity parameters and scoring model for protocol design.**

**Study parameter #**	Routine/standard (0 points*)	Moderate (1 point*)	High (2 points*)
1. Study arms/groups	One or two study arms	Three or four study arms	Greater than four study arms
2. Informed consent process	Straightforward studies with simple design	Simple trials with a placebo arm	Highly complex study to describe to potential research subjects
	Possibility for a waiver or informed consent process/informed consent documentation waiver	Studies with a separate steps for subject registration/randomization process	
3. Enrollment feasibility/study population	Study population routinely seen in clinical practice	Study population with uncommon disease/condition	Study includes vulnerable population, such as elderly, pregnant women, minority groups, terminally ill; special provisions for protection of vulnerable population and additional recruitment efforts will be required
		Study population has a common disease not usually considered for trials	Trial has highly selective eligibility criteria with required genetic/molecular markers screening
4. Subject registration and randomization process	One step	Separate process for registration/randomization	Studies involving multiple steps/randomizations or intraoperative randomization
			Complex review prior to randomization provided by centralized pathology and/or imaging vendors
5. Nature of investigational product and complexity of administration	Outpatient setting for a single modality	Combined modality for IP application	High risk for safety profile studies: biologics and/or IP with potential for increased toxicity (i.e., gene therapy, investigational bone marrow/stem cells transplant, etc.)
		Simple inpatient setting for IP application	Investigator/site personnel credentialing/training for IP application required
6. Length of investigational treatment phase	Regimens with a defined number of cycles	Cycles of treatment are not defined, individual assessments/adjustment of dosage and/or regimen are required	Extended administration of IP
	Routine or standard therapy	Long period of hormonal, chemotherapy or standard maintenance therapy, in addition to investigational agents	Experienced pharmacists preferred and additional training on handling IP
			Special quality controls for IP preparing/dispensing required (i.e., sterility, temperature, etc. controls for trials with biologics).
7. Study teams/study staff	one discipline/one clinical practice or service involvement	Leadership provided by one clinical service with moderate number of clinical practices/hospital services/staff involved	High number of medical disciplines/staff involved
	Standard clinical research team	Utilization of internal hospital services for imaging and pathology/laboratory specimen processing	Multidisciplinary teams involving complex coordination between separate practices/services within hospital
			Additional coordination with imaging and/or external pathology/laboratory vendors for images and/or laboratory specimens processing/pathology analysis
8. Data collection complexity	Standard AE/SAE reporting	Expedited AE/SAE reporting	Real-time AE/SAE reporting above and beyond that required for adverse events electronic evaluation and reporting systems AdEERs
	Prospective submission of standard regulatory updates/study reports	Retrospective submission of regulatory data	Collection of scans for end point review
	Standard case report forms	Prospective submission of a larger than normal amount of regulatory data	Central review of imaging dictates treatment decisions
		Additional data collection (e.g., concomitant medications case report forms, intraoperative data sheets, etc.)	Increased data collection requirements
9. Follow-up phase requirements	Follow-up phase data collection	Follow-up phase data collection	Follow-up phase data collection for chronic disease/indications and implantable devices
	Up to 3–6 months of follow-up upon completion of IP administration phase	1–2 years of follow-up	3–5 years or >5 years of follow-up
10. Ancillary studies A* Includes correlative pathology, imaging studies	Ancillary studies for routine pathology (i.e., cells of blood counts, clotting factors, blood chemistries, etc.)	Ancillary studies for pathology/imaging beyond routine care (i.e., additional kidney function tests are required for contrast utilization in imaging such as MRI)	Complex ancillary studies with special research protocols/guidelines for pathology/laboratory medicine services, research imaging protocols beyond routine care (i.e., research scans, tests for biological markers, study drug response monitoring, diagnostic markers for disease progression assessment, etc.)
	Modifier 1	Modifier 2	Modifier 3
Ancillary studies B** Includes health outcomes and QoL assessments	Simple ancillary studies for QoL	Ancillary studies for QoL with multiple questionnaires, assessments, pain scales, etc.	Multiple ancillary studies for QoL, healthcare utilization, socio-economical studies, etc. at different points of the study.
	Modifier 1	Modifier 2	Modifier 3

Scoring model:

Give each parameter a score of 0, 1, or 2 according to study protocol design and resources needed to conduct a study.

Parameter #10 may contribute 2 sets of complexity scores if a trial has the two different categories of ancillary studies based on the ancillary studies level of assessments referred to in this Table as Parameter #10A* (Correlative Science/Lab/Imaging) and Parameter 10B** (QoL, or health services utilization). Therefore, a protocol that has both types of ancillary studies, correlative science and QoL, has the potential to score up to 4 points (0-2 for correlative and 0-2 for QoL), depending upon which Modifier is used for ancillary studies required by protocol design.

Sum the scores of all parameters for a total complexity score and use the total scores to compare and rate trial complexity.

AE: Adverse event; IP: Investigational product; SAE: Serious adverse event; QoL: Quality of life.

**Executive summary**

**Background**

Costs associated with the implementation of clinical trials have become an increasingly important issue, yet little has been done to develop cost reduction approaches and organize efforts to improve clinical study efficiency and performance.
Increasing study protocol complexity is responsible for longer clinical trials duration, greater difficulty in recruiting research subjects and rising drug development costs. This trend is spurring new approaches to optimizing protocol design, according to leaders from the research-based drug industry.

**Assessment of study design complexity: study protocol, processes, procedures and scoring model**

Methodology of optimizing protocol design, performing complexity assessment of study protocol and procedures at planning phase of a clinical trial is described.

**Project planning: managing complexity of study protocol, ensuring billing compliance**

Approaches to determining routine costs in clinical trials based on study protocol design are discussed and guidelines for clinical trials billing compliance based on current regulations are provided.

**Conclusion & future perspective**

Application of complexity scoring model allows clinical research organizations and medical centers manage staffing objectively, ensure adherence to study protocol and procedures, effectively allocate resource and mitigate billing compliance risks via performing coverage analysis upfront and in conjunction of study protocol design and complexity.
